# *Sauropus androgynus* (L.) Merr.: a multipurpose plant with multiple uses in traditional ethnic culinary and ethnomedicinal preparations

**DOI:** 10.1186/s42779-022-00125-8

**Published:** 2022-03-07

**Authors:** Thattantavide Anju, Nishmitha Kumari S. R. Rai, Ajay Kumar

**Affiliations:** Department of Plant Science, School of Biological Sciences, Central University of Kerala, Periye, Kasaragod, Kerala 671316 India

**Keywords:** *Sauropus androgynus*, Malay cheera, Chakurmani, Chinese soppu, Ethnopharmacology, Ethnic foods, Traditional foods

## Abstract

Various plants form the basis of multiple traditional ethnic cuisines and ethnomedicinal practices across the globe. The ethnic cuisines cater to the nutritional, dietary and medicinal requirements of the tribal and rural communities even today. Using literature from various scholarly databases, this study was conducted to consolidate a comprehensive review on the use of *Sauropus androgynus* (L.) Merr. in various traditional ethnic cuisines and ethnomedicinal preparations across the globe. The survey shows that it is used in multiple ethnic cuisines and is variously known in different countries and among the communities. Further, it possesses multiple nutritional and ethnomedicinal properties. Considering its importance in ethnic foods and ethnomedicinal preparations, it is important to investigate the nutritional composition, phytochemical constitution and pharmacological basis of ethnomedicinal uses. Therefore, we further compiled this information and found that it is a rich source of both micro- and macronutrients and packed with several bioactive compounds. Survey of pharmacological studies on its traditional medicinal uses supports its ethnomedicinal properties. Despite its importance in traditional food and ethnomedicinal systems, it remains underexplored. Limited information on the toxicity of its various extracts shows that further studies should be conducted to understand its safety aspects. Further clinical studies to prospect possible drug candidates from it should be attempted.

## Introduction

**Fig. 1:**
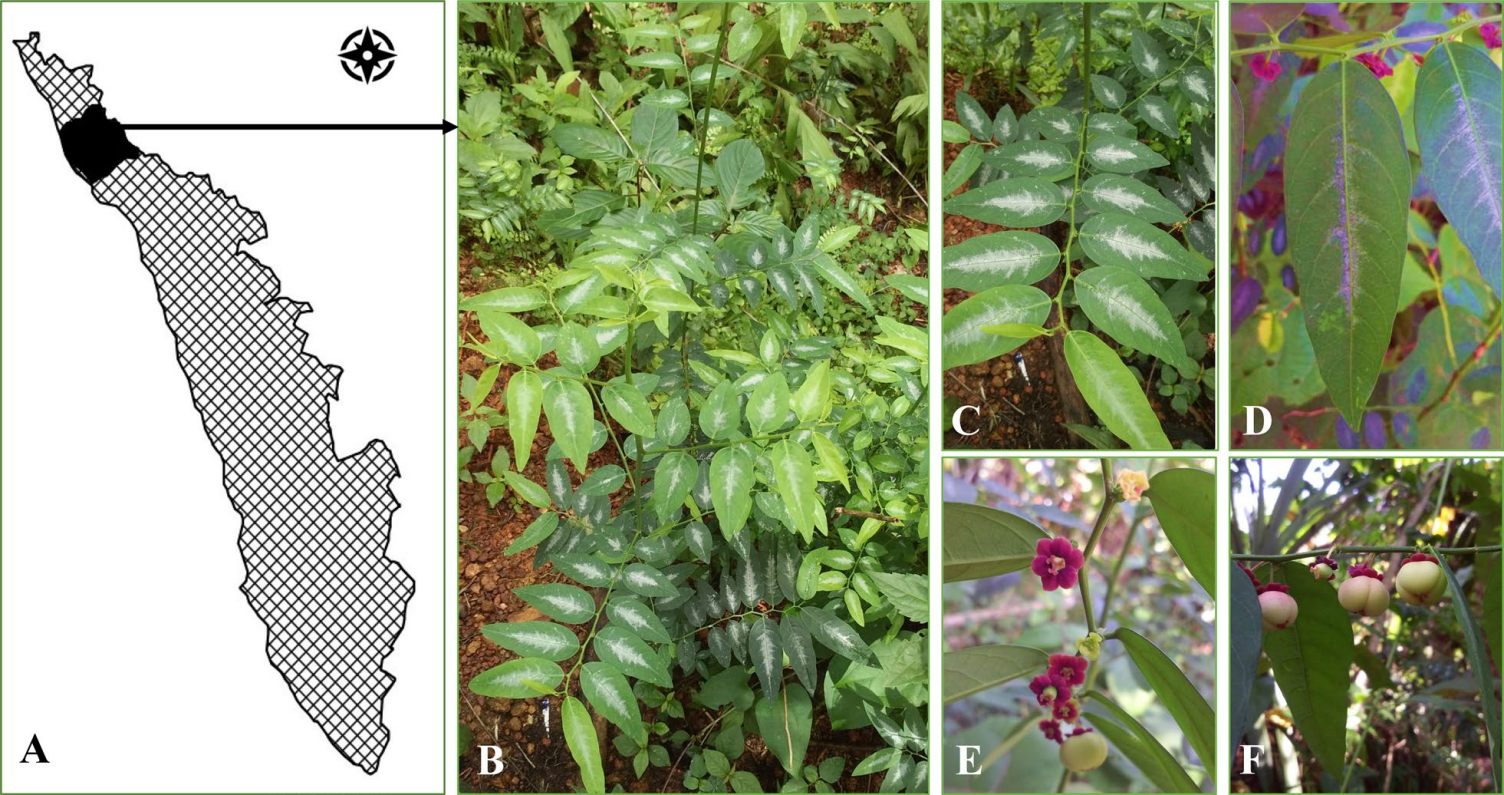
**A.** Map showing Kannur district of Kerala state of India where the photos of *S. androgynus* are captured. The plant and its various parts (**B**, **C**). **B.** Complete plant in its natural habitat, **C.** a compound leaf, **D.** a leaflet, **E.** Flower, and **F.** Fruits

People across the globe have had intimate relations with plants for food, fodder, medicines and cloths since time immemorial. Although global food systems are highly homogenised and rely on some of the major staple crops such as rice, wheat and maize, many people in different countries still use plant-based traditional ethnic foods [1, 2]. Different ethnic foods may be prepared from a single plant showing the diversity of ethnic food preparations and their biocultural significance [3]. The ethnic delicacies obtained from the plants may not be very popular globally, but they hold very high local and regional importance in a region or among a community or a society. The traditional recipes vary from one region to the other and formal documentation of preparation of ethnic cuisines may not be available. The knowledge of the ethnic preparations is orally transmitted and sustained through practice [4]. It is only recently that a large number of urban folks have also shown interest in ethnic cuisines. The plants used for the preparations of the ethnic cuisines are also often used in the ethnomedicinal preparations that form the basis of limited healthcare in the rural and tribal areas [5]. Therefore it is not surprising that some of the plants used in the ethnic dishes also possess medicinal properties. *Sauropus androgynus* (L.) Merr. belonging to the family Phyllanthaceae is such a plant with multiple uses in traditional cuisines and ethnomedicinal preparations [6]. *S. androgynus* is a shrub that grows in high temperature and humid conditions. Its branches are either cylindrical or angled. The leaves are pinnately compound with ovule or lance shape. The flowers are dark red in colour and fruits are globular in shape which is a light yellow colour (Fig. 1) [6]. The *S. androgynus* is distributed throughout the Southeast Asian mainland and Australia [7]. It is cultivated in India, Bangladesh, and Guangxi, Guangdong, Hainan, and Yunnan provinces of China [8]. Thailand–Indochina and Australia are the two important centres of diversity of *Sauropus* [7]. The findings of various studies show that *S. androgynus* is used in a number of ethnic recipes in South and Southeast Asia. Various preparation methods are used and the dishes are also known by different names.

The recent COVID-19 pandemic has exposed the fragility of long-distance interconnected mainstream food systems [9]. Disruptions in the transport sector caused due to the pandemic have resulted in food insecurity issues in several countries that heavily rely on food imports [10]. The long-term issues may further arise if the COVID-19 continues and new highly contagious variants emerge [11]. Several recent studies have pointed towards the relevance of the local traditional food systems that are locally adapted. The studies have also demonstrated that those countries or regions that have strong local/traditional food systems have shown increased resilience to the food security challenges during the current pandemic [12]. The COVID-19 has led to an increase in homestead and kitchen gardening in various countries [13, 14]. Therefore although local ethnic food systems may not be contributing to very high food security, they are very relevant under the current circumstances. The local ethnic food systems based on plants can help in strengthening the food security in those areas that are severely affected by COVID-19 in the short term [14]. The ethnic food systems are therefore important and relevant in the present context for increasing the food system resilience as well. The ethnic food systems across the globe should be studied and documented.

The phytochemical and nutritional profiling of the plants used in the traditional food and medicinal systems is crucial to understand the nutritional composition and basis of its ethnomedicinal value. The studies on the nutrient composition of plants used in ethnic cuisines provide an understanding of their relevance in the food security of the tribal and rural communities [15, 16]. The medicinal potential of a plant depends on the chemical compounds that are packed inside the plant body with reference to their quantity and quality [17]. The consumption of the plants that possess medicinal as well as nutritional properties is important considering the current health issues and the out of the pocket expenditure of the households not only in the developing countries but also in the developed world [18, 19]. Medicinal plants are an important part of the healthcare system of the consumers [18]. *S. androgynus* possess medicinal properties due to its unique phytochemical composition [20]. Various metabolites have been identified and quantified from *S. androgynus* that suggest its medicinal value [21]. Ethnobotanical studies have revealed that it is traditionally used in various formulations against a wide range of ailments including diabetes, weight loss, diarrhea, cough and ulcer [22–26]. This review particularly focuses on the usage of *S. androgynus* as traditional ethnic food and an ethnomedicinal plant in different parts of the world. Since it is an important part of ethnic cuisines, this article further explores its nutritional composition. To understand the basis of its ethnomedicinal properties, a review on its pharmacological investigation is also performed. The aim of this review is to provide a comprehensive update on the use of various species of *S. androgynus* for ethnomedicinal and ethnic foods in various tribal and rural communities.

## Traditional ethnic preparations from *Sauropus androgynus*

**Fig. 2:**
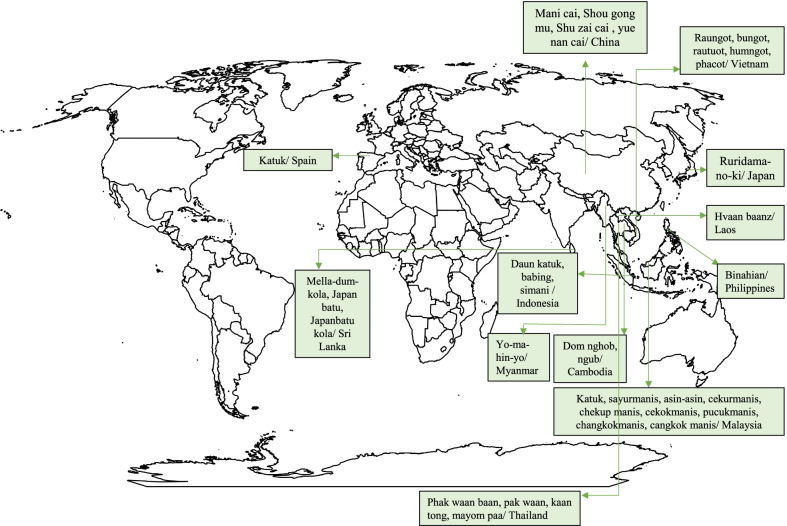
Map showing ethnic names of leafy vegetable cuisines of *S. androgynus* in various countries of the world

*S. androgynus* is widely consumed and often cultivated in south Asian countries and is adapted to high humidity and high-temperature conditions [6, 7]. It is a staple vegetable crop and originated in Borneo [27]. The taste of raw leaves is similar to peanuts and cooked leaves taste like spinach [28]. The plants are perennial shrubs with erect stems and dark green leaves. The leaves are compound leaves with a papery texture (Fig. 1) [29]. Seeds are black coloured and the plants develop fruits during July–December. It is majorly propagated through stem cuttings because of the low germination capacity of the seeds [30]. In Indonesia, more than 30 tribal communities cultivate the katuk plant especially in their home gardens and along with cabbage (*Brassica oleracea*) and beluntas (*Pluchea indica*) [31]. The vernacular names of the species include katuk, sweet leaf bush, chekusmaria, asin-asin star gooseberry [6, 32]. The plant has various ethnic names in different corners of the world. The Chinese people name the plant as Shu zai cai/ Mani cai/ yue nan cai/ Shou gong mu. In Indonesia, they are known as babing/ Daun katuk/ simani. Among the Japanese people, they are popular as Ruridama-no-ki and among Cambodians as Dom nghob. Hvaan baanz and Binahian are the vernacular names of the plant in Laos and the Philippines respectively. In the case of Malaysia, they have multiple ethnic names such as sayur manis, katuk, cekur manis, asin-asin, cekok manis, changkok manis and cangkok manis. The general name of the plant in Thailand is Phak waan baan but on the northern side, they are known as Kaan tong. Figure 2 represents the world map with ethnic names of *S. androgynus* in various countries of the world.

**Fig. 3:**
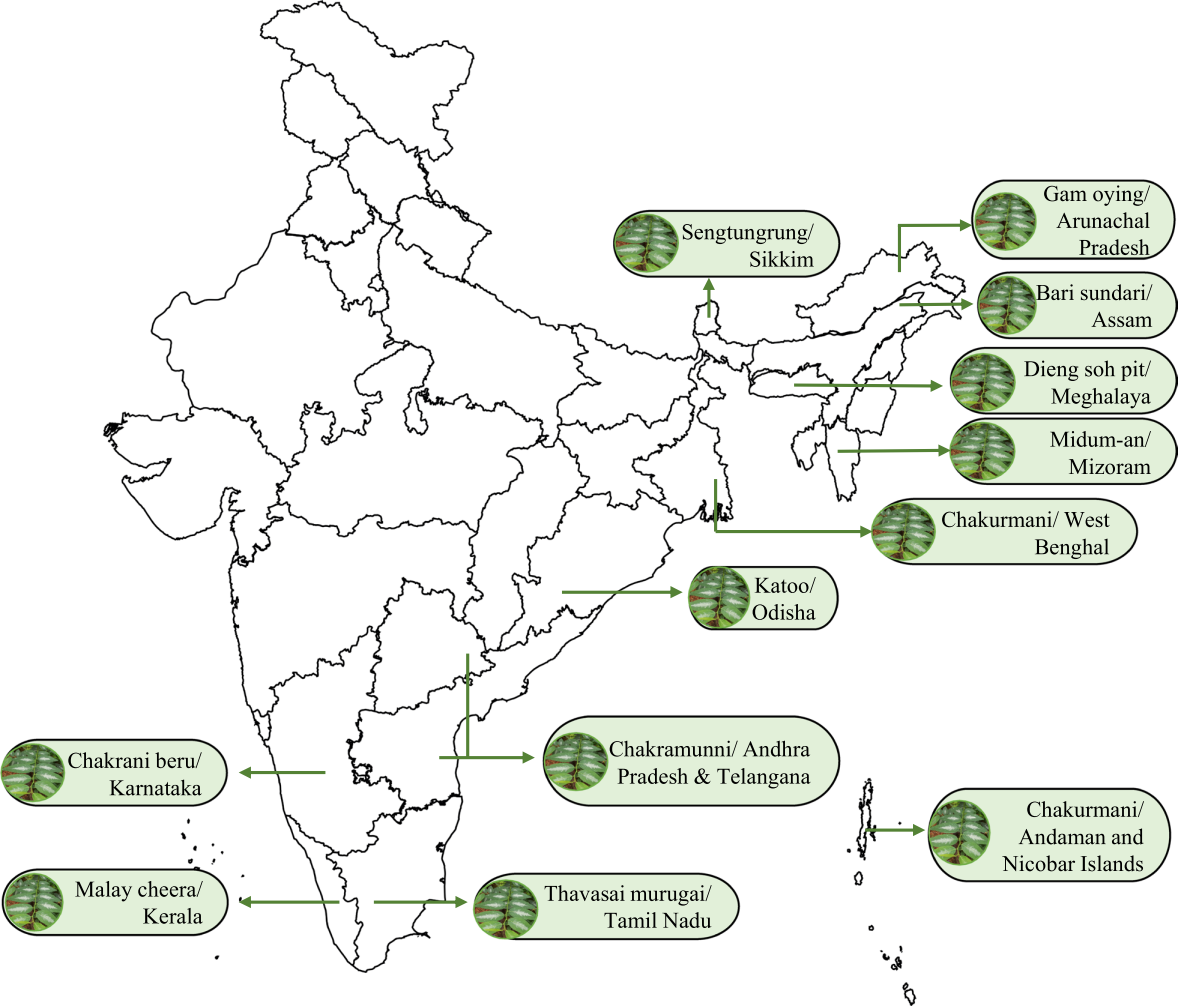
Map showing ethnic names of ethnic leafy vegetables of *S. androgynus* in various states of India

In the case of India, the plant has distinct names in distinct states including Sengtungrung (Sikkim), malay cheera (Kerala), Chakrmani (Andaman and Nicobar islands), Chakurmani (West Bengal), Chinese soppu (Karnataka) and Dieng soh pit (Meghalaya) [33]. Figure 3 shows the ethnic preparations made from *S. androgynus* from different states of India. *S. androgynus* is one species from the genus which is popular as a leafy vegetable and is used for preparing food items in different corners of the world [32]. Various consumption and cooking methods including salads, soup, curry, mixing with egg and rice, stir fry, and steam are used [32, 33]. The edible portions of the plant include young shoots, tips and leaves. In India, the Muthuvan tribes in the Idukki district of Kerala state and rural people of South Karnataka state consume tender shoots and leaves of the plant [34]. In the state of Kerala, India, it is widely consumed as a major leafy vegetable by the Malayali population. Records state that the plants were introduced to Kerala from Malaysia in 1953 hence they are popular as Malay cheera [35].

**Fig. 4:**
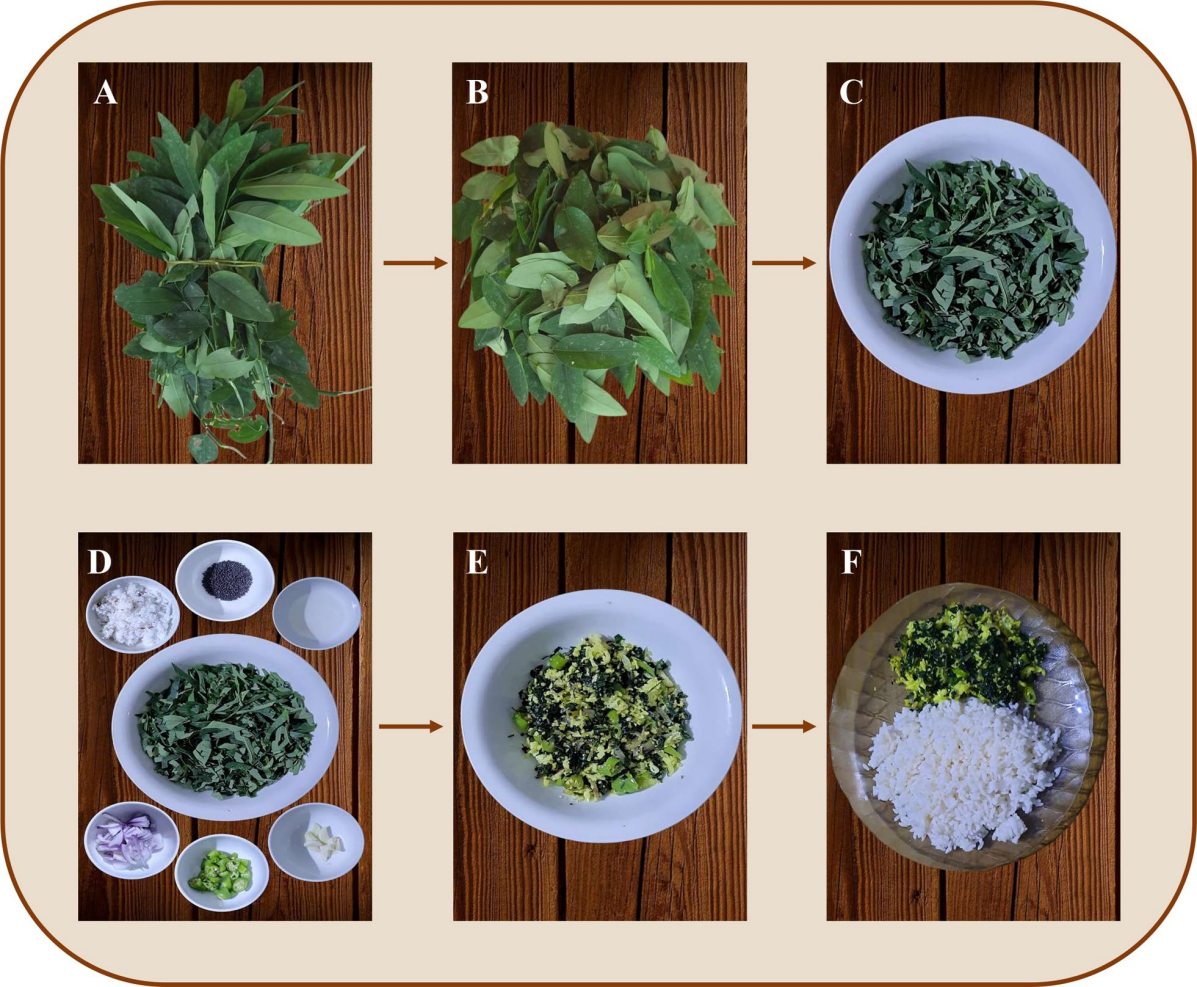
Preparation of stir-fried *S. androgynus* in Kerala. **A.** Collection of the compound leaves from the plant. **B.** Separation of the leaflets from the rachis. **C.** Chop the leaves into small pieces. **D.** Add chopped onion, chili, grated coconut and garlic for more taste and flavor. Coconut oil and mustard is used for stir-frying. **E.** Mix all the components and stir-fry them for three minutes after adding sufficient salt and turmeric powder. **F.** The delicious dish can be consumed along with boiled rice as a side dish

The general recipe of preparation of *S. androgynus* leaves in Kerala is shown in Fig. 4. The compound leaves of the plants are used for cooking. After chopping the leaves, chopped onion, chilli, grated coconut and garlic are added along with the leaves for more taste and flavour. Coconut oil and mustard are used for stir-frying. The stir-fry method is used for cooking after adding sufficient salt and turmeric powder. It is usually consumed as a side dish and served with boiled rice. The ethnic groups of Arunachal Pradesh, India also consume the leaves as cooked vegetables [36].

The young leaves and shoots are eaten by people of the Philippines in a method similar to the preparation of *Moringa oleifera* [37]. Ogle et al. [38] reported that consumption of the *S. androgynus* contributed to significant micronutrient security among the women at the time of flood and rainy season in the Mekong delta, Vietnam. People of Vietnam make soup of the leaves by mixing it with meats such as dried shrimp, crab and minced pork. At the same time, in Malaysia, it is commonly stir-fried with eggs and dried anchovies [33]. In Japan, the leaves and short tips are exported as tropical asparagus [33]. In Malaysian multi-racial cultures, these vegetables are usually eaten raw as a salad which is known as a cekur manis and ulam [33]. The other species that are consumed as vegetables are *S. bacciformis, S. macranthus, S. rhamnoides, S. thorelii* in Southeast and East Asian countries such as Thailand and China [12]*.* The leaves and mericarp of *S. macranthus* are edible [12]. The fruits and leaves of *S. rhamnoides* are consumed in Thailand [7]. Traditional modes of preparation and consumption of the edible part of genus *Sauropus* are given in Table 1.

**Table 1:** Traditional food systems that are based on the *S. androgynus*

Country/State/Region/Tribal/indigenous community	Part used and mode of preparation	Reference(s)
*S. androgynus*		
Natives of Ilocos Norte, Palawan and South Central Mindanao-Philippines	Young leaves and shoots are cooked	[37]
Indigenous community in the Solomon Islands, northern Australia	Curry is prepared from their leaves	[57]
Villagers of Mekong Delta, Vietnam	Consumed as leafy vegetables	[38]
Throughout Thailand	Shoot, fruits and leaves are eaten raw and cooked. Soup is also prepared	[7]
Malaysia	Leaves are eaten raw and sometimes included in salads also	[33]
Karbi tribes in Assam, India	Curry is made with leaves	[58]
Muthuvan tribes In Kerala, India	Tender leaves are cooked and consumed	[34]
*S. macranthus*		
Southeast Asian countries	Leaves and mericarp are consumed	[7]
*S. rhamnoside*		
Thailand	Fruits and leaves are consumed	[7]
*S. thorelii*		
Endemic to Laos	Leaves are cooked and consumed	[7]
*S. spatulifolius*		
South China Guangdong region	Herbal tea is prepared with an aqueous decoction of the whole plant	[59]

It is not only popular for its nutritional properties, but also for its therapeutic potential [39–43]. It is used to cure several ailments such as fever, urinary problems, ulcers, pain relief, malaria and interestingly they are used to increase the production of breast milk in feeding mothers in various corners of the world [24, 44, 45]. In South Asian countries, it is used as a slimming agent which indicates its anti-obesity activity [46]. *S. androgynus* have several phytochemicals such as resins, saponins, flavonoids, glycosides, catechol, acidic compounds, tannins, alkaloids, sterols, terpenoids, phenols and cardiac glycosides [47, 48]. Besides nutritional and therapeutic potential, this plant is reported to contain 18–20% of fatty acids which suggest its potential to be used as biofuel feedstock [49]. The ability of *S. androgynus* to grow in heavy metal-containing soil points towards its ecological importance of phytoremediation [50].

Few other species of *Sauropus* such as *S. androgynus, S. bacciformis, S. compressus, S. macranthus, S. rhamnoides* and *S. thorelii* are also used for medicinal and nutritional purposes. *S. bacciformis* is a subshrub or herb which grows up to the height of 60 cm tall, monoecious, erect, prostrate or diffuse and glabrous entirely, solitary stems, and several branches from the base [51]. Indian people use this multivitamin plant to cure pyrexia and to treat diseases related to the urinary system [52]. *S. bacciformis* L. is used for indigestion, leaves of this plant ground along with *Piper betle* and then orally administered to children for 2 days [53]. Fresh leaves of *S. compressus* Müll. Arg. is used to treat retained placenta, and mouthwash is prepared from the fresh leaves with honey [54]. Leaves are used as poultry and cattle feed [55]. It is also used as an ornamental plant [56]. *S. macranthus* Hassk. is also edible, but it is the least popular species in the genus. Its leaves and mericarp are edible. It is used as an ornamental plant in Java [7]. *S. rhamnoides* Blume is edible and found in Thailand, Malaysia, India, Sumatra and the Philippines. Its fruits and leaves are consumed [7]. The *S. thorelii* Beille is endemic to Laos and its leaves are used for cooking and flowers are used for fragrance [7].

## Nutritional value

### Nutritional components

The studies on nutritional composition help to reveal the nutritive capacity of edible plants. Extensive research on various major and minor nutrients are required to screen the nutritional profile of a plant. Exploration of edible value of the plants from the genus *Sauropu*s is comparatively less and majority of the research is centralised on *S. androgynus* species*. S. androgynus* is also called a ‘multigreen vegetable’ because of its high nutritional value and vitamin quantity in comparison with other vegetable crops [6, 60]. *S. androgynus* is a rich source of vitamin A and C, protein, calcium and carbohydrates in comparison with other leafy vegetables such as amaranth and drumstick leaves [61]. It has been proved that the concentration of water-soluble vitamins is higher in *S. androgynus* leaves than fat-soluble vitamins [62]. The fully matured leaves of *S. androgynus* are recognised as a rich source of β-carotene. Studies reveal that they are also rich in fat-soluble vitamin E which have antioxidant properties [62]. A comparative study was performed to understand the difference between the nutritional composition of basal whorl leaves and terminal whorl leaves of *S. androgynus* by Naveena et al. [61] and found that basal whorl leaves are more enriched with nutrients than terminal whorl leaves. They also reported that there was a significant elevation in the quantity of nutrients such as proteins, carbohydrates, calcium and vitamin C from day 60 to day 120 which suggests the difference in nutrient accumulation inside plant organs according to their developmental stages [61].

Elemental composition studies showed that fully matured leaves of *S. androgynus* have elevated quantities of iron and zinc [62]. Calcium content of fully matured leaves is also higher than the tender leaves. Iron is an abundant element found in this plant and has a high concentration that reaches greater than 100 mg/kg [63]. The investigation by Santoso et al. [64] shows that the addition of turmeric and garlic to fermented *S. androgynus*-bay leaves enhanced methionine, arginine, tyrosine, aspartic acid, histidine, valine and total amino acid in broiler chicken meat. They hypothesised that different sulphur compounds (S-allyl cysteine, allicin, diallyl disulfide) diversity of garlic can induce insulin production, which results in increased uptake of blood amino acids to muscle. It also induces the synthesis of methionine by bacteria in the stomach. They also suggest that the synergic interaction between turmeric and garlic along with fermented *S. androgynus* increased the amino acids such as arginine in the meat of broiler chicken because individual supplementation of these things did not make any impactful changes in the amino acid composition inside the organism. The study proved the possibility of increasing the nutritional value of food supplements with the combination of such traditional plants and plant-derived products. Detailed nutritional composition of *S. androgynus* with their biological activities is presented in Table 2.

**Table 2:** Nutritional composition of *S. androgynous*

Constituents	Quantity (references)	Function (references)
*Macronutrients*		
Proteins	10.83% [65]; 15.8 g/100 g [66]; 5.2% [20]; 3.04% [67]; 8.31 g/100 g [68]; 7.4 g/100 g [6]; 15.0% [64]	Obesity control by elevating high-density lipoprotein (HDL) cholesterol [69]
Carbohydrates	54.5 g/100 g [66]	Carbohydrates in our body maintain the energy potential [70]
Chlorophyll	14.43 ± 0.16 μg/mL [71]	Antioxidant agent [72]
Fatty acids	62.92% [65]	Act as an energy donor complex, interconnected systems [73]
Crude fibre	1.87% [65]; 36.0 g/100 g [66]; 1.75% [20]; 1.07% [67]	Enhances food digestion [70]
Crude fat	0.85% [65]; 0.58% [20].; 4.0 g/100 g [66]; 1.07% [67]	Helps in fat-soluble vitamin absorption [70]
*Micronutrients*		
Fe	8.8 mg/100 g [32]; 13.5 mg/100 g [74]	Participate in oxygen transport [75]
Zn	15.9 g/100 g [76]	Growth, development and defense [77]
Mn	664.9 mg/100 g [33]	Plant growth and development [78]
Cu	768.7 mg/100 g [76]	Ensure cellular functions [79]
Mg	664.9 mg/100 g [76]	Activates enzymes [80]
K	45.7 mg/100 g [76] 45.70 mg/100 g [20]	Cofactor that functions in protein synthesis [80]
P	543 mg/100 g [32]	Major constituent in phospholipids, nucleic acids, adenosine triphosphate (ATP), coenzymes [80]
Ca	2.8% [74]; 118.8 mg/100 g [74]	Important information and stability of cell walls and in maintenance of membrane structure and permeability [80]
Co	1.62 mg/100 g [76]	Integral part of the structure of vitamin B12 (Cobalamin) [81]
Vitamin A	4.11 mg/100 g [61]	Cancer prevention [82]
Vitamin C	244 mg/100 g [32] 56.1 mg/100 g [74]	Essential for mental and physical development of the body [33]
Vitamin E	17.8 mg/100 g [62]	Inhibition of platelet aggregation [83]
Carotene	5600 μg/100 g [32]	Reduce cancers and eye disease risk [84]

### Bioaccessibility of nutrients

The cooking procedure induces significant alterations in the nutritional, sensory and structural composition of various food components [85]. The bioaccessibility of the nutritional components is highly dependent on the cooking methods [86]. *S*. *androgynus* is a good source of β-carotene (5.6 mg/100 g), but Padmavathi and Rao [32] reported the degradation of protein and beta-carotene at the time of heating of their leaves. Arumsari et al. [87] investigated the influence of various cooking methods, such as microwave cooking, boiling, and raw consumption and palm oil addition on the bioaccessibility of β-carotene from *S. androgynus.* They reported that the β-carotene content in microwave digested and boiled leaves were less than in raw leaves, but the addition of palm oil increased bioaccessible β-carotene content in all cooking methods tested by them. Azima et al. [88] investigated the influence of different cooking methods on the phenol, vitamin C, Fe, Mg and Zn content of *S. androgynus* leaves. This study reported that stir-frying cooking increased the content of Zn, Mg, Fe and phenol in the leaves, while it reduced vitamin C content. The boiling and steaming method resulted in the reduction of these components. From the studies, it is clear the mode of consumption or mode of cooking have a crucial role in the contribution of nutrients to consumers from the plant.

### Food fortification with *Sauropus androgynus*

Food fortification is an important method to enrich the foods deficient in a particular nutrient. The method of food fortification is helpful for the reduction of micronutrient deficiencies of the populations effectively [89]. *S. androgynus* have been used for the fortification of staple food [90, 91]. Hasrini et al. [90] explored the possibility of nutritional fortification of cassava flour cookies with *S. androgynus* along with *M. oleifera* and *Brassica oleracea*, because of lack of minerals, fat and protein content in cassava flour modified cookies. Fortification of the cookies with these vegetables enriched the nutritional profile of the cookies more than pure cassava flour. Among them, cookies fortified with *S. androgynus* displayed high mineral content (Ca, K, Mg) after *B. oleracea* fortified cookies. Similarly, sago noodles are also a carbohydrate-rich staple food in the area of Riau Province, Indonesia. But they lack other essential nutrients such as proteins. Research by Dewita et al. [91] attempted to fortify the sago noodles with fish oils, fish proteins. *S. androgynus* was also included as a vegetable source of vitamins and fibre. Finally, they reported highly nutritional rich noodles compared to raw sago noodles. Further studies must be conducted to fortify the foods with *S. androgynus*.

## Ethnomedicinal importance of *Sauropus androgynus*

Plants play an important role in the life of humans in all stages of their life span [92]. Our ancestors have been dependent on plants for their various therapeutic uses since time immemorial [93]. The plant-derived medicinal formulations are used against various primary disorders by different populations around the world [93]. The initial stage of drug discovery is the documentation of traditionally used medicinal plants and other materials [94]. But the knowledge regarding traditional medicine is vanishing from generation to generation due to the development of modern treatment methods and due to the influence of urbanisation [94]. Therefore investigation of traditional ethnomedicinal knowledge of plants is very important and it can help to promote the conservation of the plants and provide an opportunity to validate the medicinal use [95]. *S. androgynus* possesses several therapeutic uses [76]. *S. androgynus* plays a major role in traditional medicinal systems for curing some ailments as it is a good source of fatty acids, polyphenols, and flavonoids [20]. Lee et al. [96] investigated the importance of *S. androgynus* in the Chinese herbal medicinal system and reported that the villagers depend on the treatments of various ailments such as laryngitis, cough, hepatitis, constipation, blurred vision and enteritis. Besides *Plectranthus amboinicus*, *S. androgynus* is used to increase breast milk production [97]. In Taiwan, people use this plant as a slimming agent to tackle obesity [6]. 3-O-β-D-glucosyl-(1-6)-β-D-glucosyl-kaempferol (GGK) is the chemical compound found in *Sauropus* plants which can act as an antiobesity agent [98]. *S. androgynus* increases the fat content of milk, therefore providing *S. androgynus* leaves along with gamal leaves to cows can improve the fat content of the cow milk [99]. In another study, it was reported that the leaves of the *S. bacciformis* are used for indigestion, especially in children. The leaves are ground along with *Piper betle* and then orally administered for 2 days for proper digestion [53]. Fresh leaves of *S. compressus* are used for treating retained placenta [54]. The ethnomedicinal uses of *S. androgynus* in different countries of the world and different states of India are shown in Tables 3 and 4 respectively. More ethnomedicinal studies are required to screen the ethnomedicinal uses of *S. androgynus* from other parts of the world. The validation of its reported ethnomedicinal properties should be performed through pharmacological experiments to obtain scientific and reliable evidence.

**Table 3:** Ethnomedicinal uses of *S. androgynus* across different countries

Country/state/region/district	Common name	Part(s) used	Ethnomedicinal use(s)	Mode of usage	Reference(s)
*Asia*					
Indonesia	Katuk	Leaves	Uterotonic agent	Fresh leaves and roots	[20]
			Febrifuge	Leaves are blended, then put on the head	[100]
			Cough	Leaf extract is mixed with kencur (*Kaempferia galanga* L.) water and then drunk	[100]
	Kayu manis, daun katuk, katuk	Leaves	Heartburn, and for cleaning the blood	Leaf decoction	[101]
Malaysia	Sayur Manis	Root	Fever and urinary bladder complaints	Root decoction	[20]
India	Star gooseberry, Chinese soppu	Root	Diarrhea	Root powder	[102]
Thailand	Phak wan ban	Root, leaf	Aphthous ulcer	Boiling to drink	[103]
China	Shu zi cai	Leaf	Cough	Leaf extract	[20]
Vietnam	phac ot	Leaf, stem and root	fever		[104]

**Table 4:** Ethnomedicinal uses of *S. androgynus* in different parts/states of India

States	Tribal/ community/ sub area	Common name	Part(s) used	Ethnomedicinal use(s)	References
Andaman & Nicobar Islands		Chakarmani	Leaf	Vision and skin problems	[55, 76]
Assam	Sonowal Kacharis	Bari sundari	Root	Tongue ailment (Appearance of white layer on the tongue of children)	[55, 105]
Karnataka		Chakrani beru, Chinese soppu	Root	Maceration of root with lemon juice and then applied on the bite (snake bite)	[55, 106]
	Soliga tribes	Chikrumani	Stem, leaves	Used to treat diabetes, inflammations and cough	[55, 106]
Kerala	Muthuvan tribes of Idukki district	Malay cheera, elacheera	Whole plant	Increase lactation	[55, 107]
Tamil Nadu	Thavasi Murungai	Thavasi murungai	Root	Decoctions for treating urinary complaints	[55, 108]
Arunachal Pradesh	Adi- Minyong tribe	Woein	Leaves	Leaf decoctions for revitalizing agents, cooked as vegetables	[109]
Mizoram	Mizos	Midum-an	Leaves	Revitalizing agent	[110]

## Bioactive compounds found in *S. androgynus*

Bioactive compounds present in plants are important for human health. They are very essential for the human body as they have several bioactivities such as anti-inflammatory, antidiabetic, antioxidant, and antimicrobial activities [111]. Specialised metabolites (earlier known as secondary metabolites) are the major phytochemicals that contribute to the bioactivity of plants [112]. Plants are the biggest source of medicinally important compounds crucial for discovering novel products and improving drug development. Plant specialised metabolites have gained considerable attention due to their potential for flavours, food additives and pharmaceuticals [113]. Functions of secondary metabolites include inhibition or stimulation of defence and microbial interactions, catalytic activity, signalling and act as structural compounds in various mechanisms in the cell [114]. A variety of specialised metabolites including steroids, flavonoids, fatty acids, alkaloids, tannins and resins are found in plants [113]. Plenty of articles are available on the richness of bioactive phytochemicals viz steroids, terpenoids, tannins, alkaloids, phenols, flavonoids, volatile oils and fatty acids in the *S. androgynus* [47]. Besides major carbohydrates, proteins, lipids, alkaloids, terpenoids and phenolics, they reported important medicinal components such as 1,14-tetradecanediol (antimicrobial activity); 1-octadecyne (antibacterial and anti-inflammatory); 1-hexadecyne (antibacterial); decanoic acid, ethyl ester (nematocide); phytol (anticancerous); 2(1H) naphthalenone, 3,5,6,7,8,8 a-hexahydro 4, 8a-dimethyl-6-(1- methylethenyl) (anti-inflammatory); azulene, aoctahydro-1,4-dimethyl7-(1-methylethenyl)-, [1- methylethenyl) and squalene with several pharmacological properties.

Andarwulan et al. [68] showed that among 11 selected Indonesian vegetables, *S. androgynus* showed the highest amount of flavonoid content, which is an indication of the antioxidant property of the plant. The hydroxyl group in the flavonoid compound facilitates free radical scavenging activity and helps in the induction of antioxidant defence mechanisms inside the human body [115]. The compounds are capable of stimulating the human protective enzyme system [115]. Besides antioxidant activity, the compounds perform inactivation of cell transport proteins, adhesins and enzymes of microbes, hence they display antimicrobial activity [116]. They are effective against viral enzymes too, hence they show antiviral activity [117]. Flavonoids are inhibitors of the phosphodiesterase enzyme (an enzyme that is involved in cell activation), and they decelerate the inflammation procedure [115]. Flavonoids are well known for retarding the action of several carcinogenic conditions [118]. Since the plant *S. androgynus* contains a good amount of flavonoids, they promise their effectiveness in various pharmacological activities mentioned above.

The detailed and prime metabolic fingerprinting of *S. androgynus* was performed by Yunita et al. [21] using Gas Chromatography–Mass Spectroscopy (GC–MS) on the leaf methanol extract from six geographical regions. Their study found wide variation in the composition of metabolites and further revealed that major portion of the *S. androgynus* metabolites is dominated by various fatty acids such as palmitic acid, myristic acid, methyl-stearate and methyl-linoleic subsequently followed by isophytol and phytols. The plant-based fatty acids are effective against the reduction of cardiovascular diseases by modifying the blood lipid profile and activating several anti-inflammatory pathways. Plant-based fatty acids interact with the gut microbiome and translocate the lipopolysaccharides too [119]. The same study revealed the presence of vitamin E in a comparatively higher quantity in the samples. Plants usually synthesise ∝ , β, γ, ẟ tocopherols from homogentisic acids and these compounds are excellent in free radical scavenging activity [83]. They display anticancer activity by activating p53 tumour suppressor gene and heat shock proteins. Vitamin E also downregulates the expression of mutated p53 proteins and has anti-angiogenic activity by blocking the transformation of alpha growth factors [120]. It was reported that vitamin E is capable of boosting the human immune system by enhancing phagocytic activity and cellular immune responses [83]. Since *S. androgynus* is rich in vitamin E, they can be included in the food system to fortify the diet of deficient people. Because vitamin E deficiency can cause problems related to the immune system, vision, muscle power and body balance [83]. Similarly, a metabolomic study done with Fourier Transform Infra-Red spectroscopy (FTIR) revealed that *S. androgynous* leaves contain carboxyl, alkene, amine salt, sulfone, amines and alkyl aryl ether [56].

The non-narcotic alkaloid compound papaverine was reported in *S. androgynus* plants. The compounds are effective inhibitors of phosphodiesterase and are used for the treatment of erectile dysfunction and vasospasm. The compound is also recognised for its anticancer activity [121]. The papaverine content in *S. androgynus* leaves is reported to increase oxytocin and prolactin production, which are the two main hormones in milk production [122]. The experiments involving mice supplemented separately with younger and mature leaves of *S. androgynus* revealed that concentration of oxytocin and prolactin increased when mice were supplemented with mature leaves of *S. androgynus* [122]. The quantity of papaverine present in the fresh leaves of *S. androgynus* is 580 mg/100 g which has the capacity to act as an antispasmodic drug [32]. In a human-based study, it has been proven that the leaf extracts of *S. androgynus* plants boost the mothers’ breast milk production [55], this can be because of the presence of papaverine in the leaves.

A new steroid 20-hydroxyisofucosterol (stigmasta-5,24(28)-diene-3β,20β-diol) was reported by Zhang et al. [123] from *S. androgynus*. The pharmacological studies of the extract showed that they have moderate levels of cytotoxic activities and it can inhibit the activity of the alpha-melanocyte-stimulating hormone. Alpha-melanocyte hormone has a crucial role in the control of several metabolism [124]. Recently Huong et al. [125] discovered three new glycosides viz. aurobaccioside A, saurobaccioside B, saurobaccioside C from the whole plant of *S. bacciformis* and displayed significant cytotoxic activity against cancerous cell lines. Another bioactive compound reported from two *Sauropus* species was eudesmin. Sawasdee et al. [126] reported eudesmin from the leaves of *S. thorelii* and *S. bicolor*. Eudesmin is popular for its antitumor, anti-inflammatory and anticonvulsant properties [127, 128]. Similarly, a novel component named sauropurostratic acid was reported from the plant *S. rostratu* by Wei et al. [129]. In the same study, they have extracted and identified a total of 19 other compounds and β-sitosterol, niacinamide, quercetin, mannitol, aurantiamide acetate and kaempferol were reported for the first time. The β-sitosterol has hypolipidemic activity since it has structural similarity with cholesterol and acts as a competitive inhibitor for cholesterol during absorption [130]. Niacinamide is an amide of vitamin B3 and is effectively used for the treatment of skin pigmentation related problems. It acts by blocking the migration of melanosomes from melanocytes to keratinocytes that suppresses skin pigmentation [131]. The phytochemical quercetin belongs to the group flavonoids and it also shows functions similar to flavonoids as we discussed earlier. The compound mannitol is a harmless natural sweetener in comparison with glucose and sucrose. Kaempferol is another antioxidative polyphenol with anticancer activity. Kaempferol modulates several proteins related to inflammation, angiogenesis, apoptosis and metastasis [132]. Wang et al. [133] detected three new hexose carbohydrate derivatives from *S. rostratu* plant, namely, Butyl 3,6- anhydro-2-deoxy-b-D-arabino-hexofuranoside, Butyl 3,6-anhydro-2-deoxy-b-D-glucofuranoside and Methyl (1R,3R,4S,5R)-3,6-anhydro-2-deoxyhexofuranoside. These are novel compounds detected from the plants and more extensive studies are required about these compounds. The detection of the bioactive compounds strengthens the validation of bioactivities of *S. androgynus* plants and provides evidence for their ethnomedicinal properties. Phytochemicals present in *S. androgynus,* with their quantities and reported biological activities are shown in Table 5.

**Table 5:**
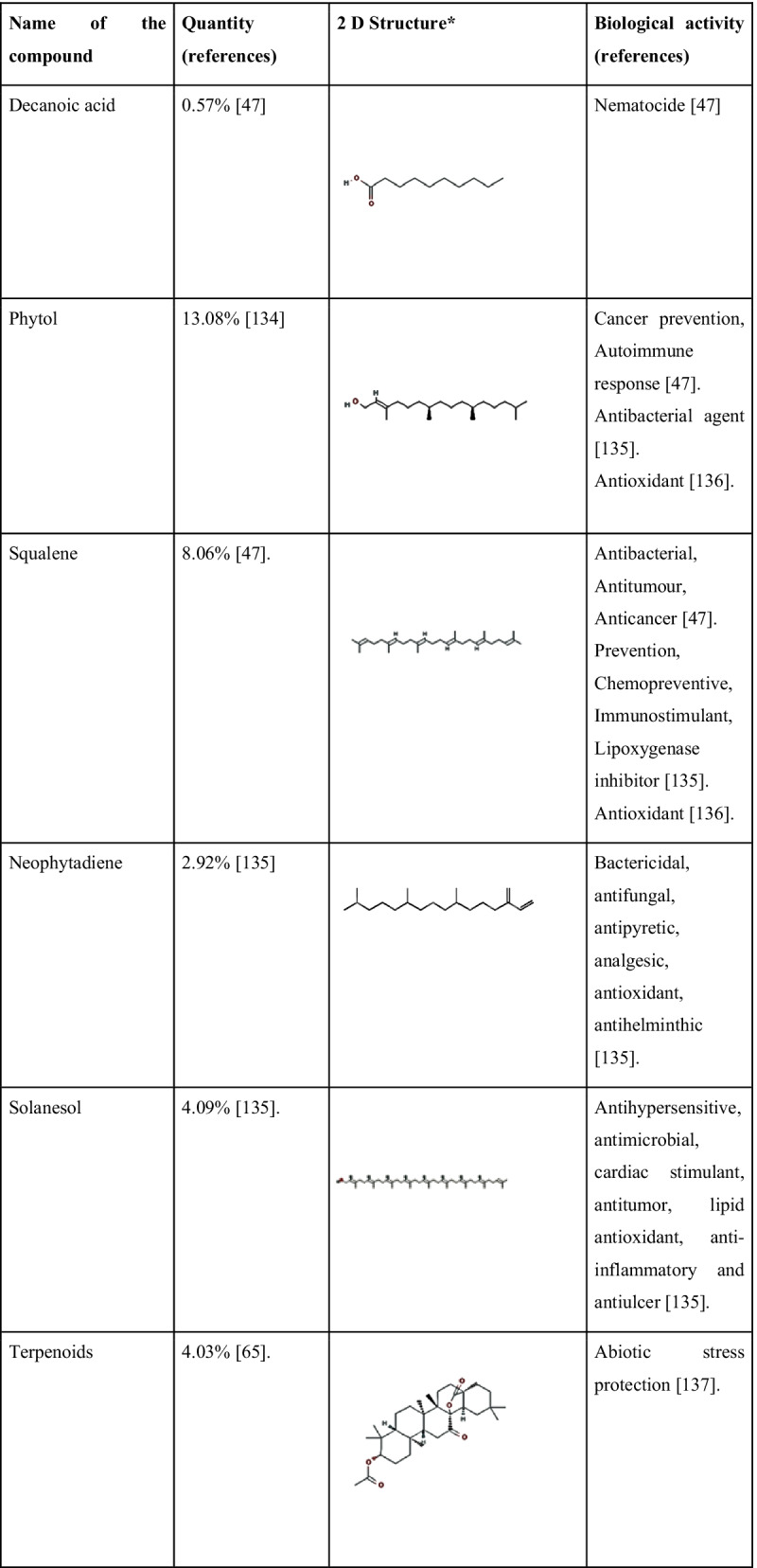

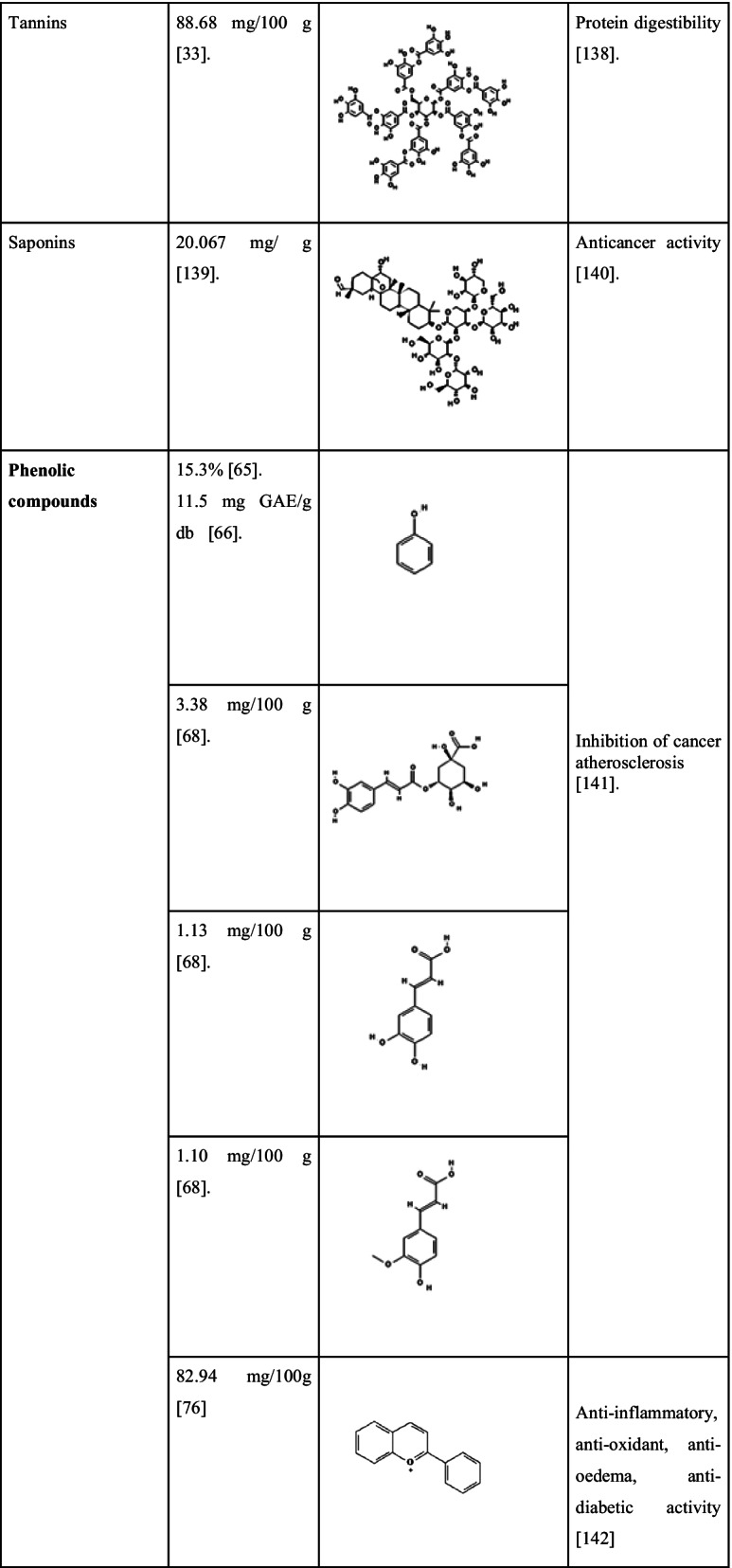

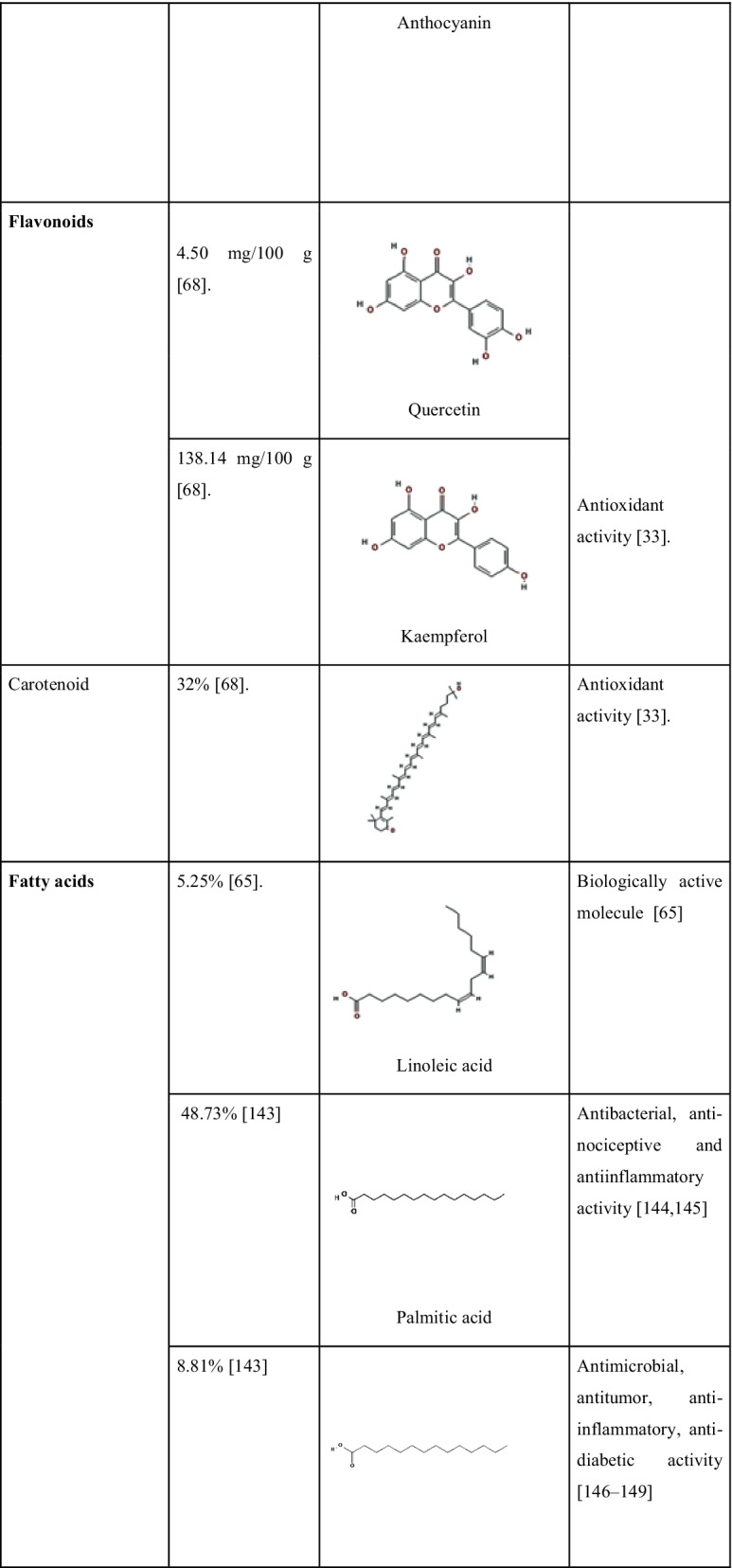

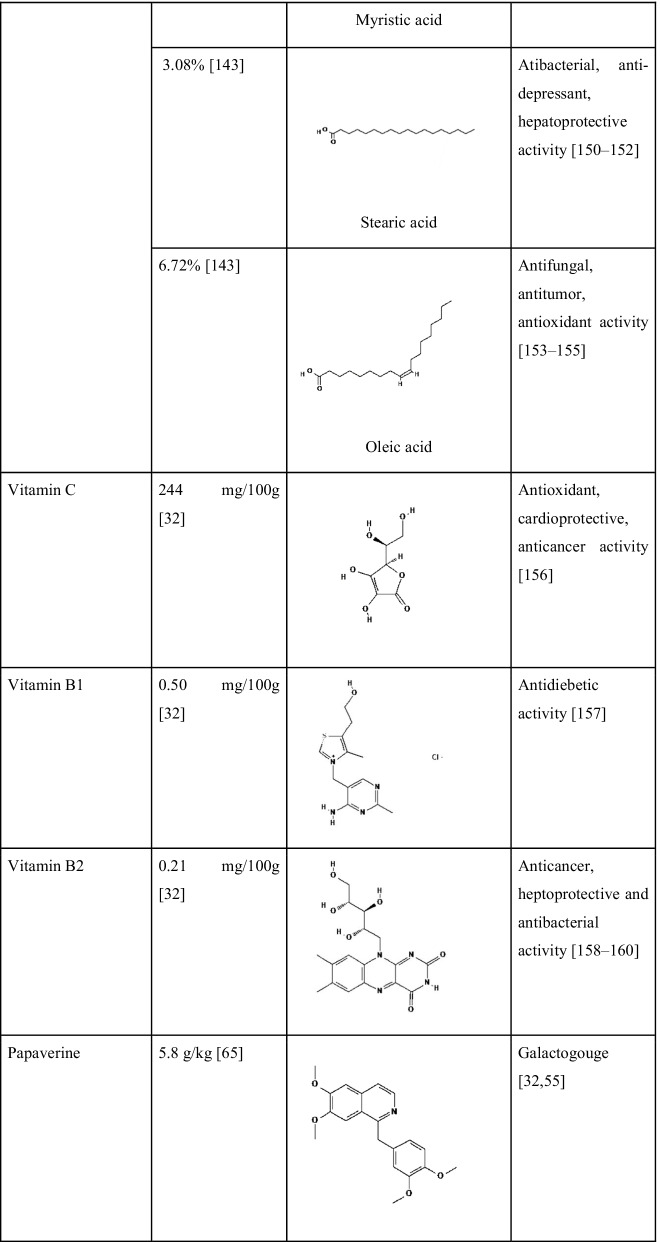
Main phytochemicals found in *S. androgynus* with their reported medicinal properties

*2D structures are taken from PubChem (https://pubchem.ncbi.nlm.nih.gov/)

## Pharmacology of *Sauropus androgynus*

**Fig. 5:**
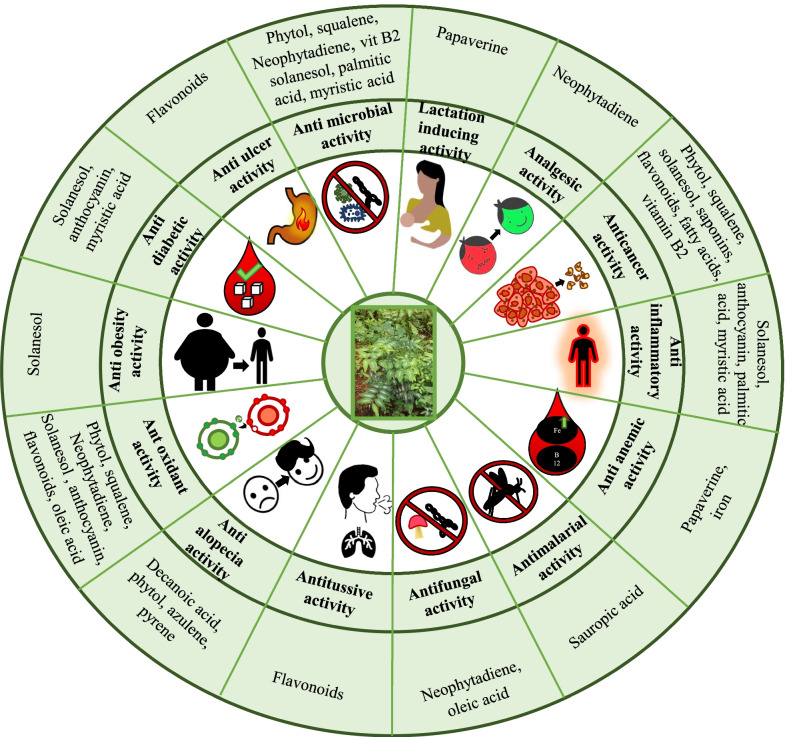
Various bioactivities and bioactive compounds of *S. androgynus*

Ethnomedicinal uses of plants are nowadays increasingly validated using pharmacological studies involving various in vivo and in vitro models [161]. As discussed in the previous section, *S. androgynus* contains some of the very important phytochemicals with proven medicinal properties. The medicinal plants exert their bioactivities through the bioactive compounds present in them. The extraction method, dosage and mode of application is very crucial in determining the efficiency of the treatment which can be achieved through pharmacological studies [162]. Some of the ethnomedicinal properties of *S. androgynus* are also recently validated using modern pharmacological studies. Pharmacological studies are also crucial for the evaluation of toxicity or potential side effects of any extract consumed by the people for the treatments of their diseases. Several researchers have reported multiple bioactivities of *S. androgynus* using pharmacological studies [163–167]. Various bioactivities of *S. androgynus* are discussed in the subsequent sections and are summarised in Fig. 5.

### Antioxidant potential

Antioxidants aid in the neutralisation of free radicals and reactive oxygen species (ROS) inside the cell [168]. Exposure to pollutants, sunlight, UV radiation, X-rays, smoke (both tobacco and automobile exhaust), and ozone by humans can result in the generation of reactive oxygen species [169]. They are highly reactive and unstable and cause damage to the nucleic acids, lipids, proteins and carbohydrates in the cell, which consequently results in the development of degenerative diseases [170]. Therefore the proper spatial and temporal elimination/ neutralisation of ROS is crucial in the day to day life of humans, which is carried out by the antioxidant compounds [169, 170]. The plant-based antioxidants are the best and most popular as they scavenge the free radicals inside the cells effectively and boost the defence mechanism by the endogenous antioxidants [171]. It was proved that supplementation of exogenous plant-based antioxidants are effective for the treatment of diseases due to ROS and it boosts the endogenous free radical scavenging machinery [172]. Antioxidants are effective as anti-ageing and anti-inflammatory agents. These compounds have multiple applications in different areas [173]. Antioxidants are added to many food items for enriching the health benefits of the diet [173]. Natural antioxidants are found in foods and prevent the sourness, disruption and colour change of the food [173]. They have high stability and low volatility, and a significant role in maintaining the level of nutrients, colour, taste, texture, freshness and aroma [174]. The common natural antioxidants present in plants include vitamin C, chlorophyll a, chlorophyll b, carotenoid, flavonoids and polyphenols [173]. Carotenoids are essential for the human body since they cannot be synthesised [33]. Flavonoids are important dietary antioxidants that are well known for their role at different developmental stages of malignant tumours by protecting DNA. It inactivates carcinogens and inhibits the mutagenic gene and enzyme expression [33]. Polyphenols are important metabolites in nature. They have protective effects on human carcinogenesis, memory and cognitive function, then some other effects such as age-related neurological dysfunctions, and ulcers [33]. Vitamins are very important for the common metabolic functions, immune responses and mental and physical development [33]. Various in vitro assays such as DPPH radical scavenging, ABTS radical scavenging, FRAP helps to understand the antioxidant activity of plant extracts which is helpful in preventing various oxidative stresses [175].

*S. androgynus* shows antioxidant properties and is rich in flavonoids which is one of the important natural antioxidants [68, 136]. According to the study conducted by Ermi Hikmawanti et al. [176], *S. androgynus* ethanolic leaf extract (50%) shows the best and potential antioxidant activity. Pasta prepared with *Sauropus* leaves reduces lipid peroxidation [177]. Polyphenols from leaf extract of *Sauropus* are also a good source of free radical activity and can be used to treat diseases developed through the action of free radicals [134]. Studies conducted by Nguyen et al. [71] showed significant 2,2-diphenyl-1-picryl-hydrazyl-hydrate (DPPH) scavenging activity of the chlorophyll extracted in 90% acetone from the *S. androgynus* plant. The experiment conducted by Badami and Channabasavaraj [178] also displayed antioxidant properties against the ABTS free radicals.

Besides flavonoids and polyphenols, *S. androgynus* is rich in Coenzyme Q10 (CoQ10) which acts as a strong antioxidant. Kettawan et al. [42] analysed the plasma antioxidant activity of *S. androgynus* leaf powder in male Wistar rats and checked the absorption and quantity of CoQ10 after the stir-frying the leaves. The study reported that the stir-frying procedure does not affect the quantity of CoQ10 in the plants and showed significant free radical scavenging activity in the ferric reducing antioxidant power assay (FRAP), oxygen radical absorbance capacity and DPPH free radical scavenging method. The antioxidant activity of tissue cultured shoots of *S. androgynus* was explored by Petchang [179]. They have accelerated the antioxidant activity with the treatment of ultraviolet C radiation and triggered the production of secondary metabolites by the addition of 6-benzyladenine in the Murashige and Skoog medium. They have observed strong antioxidant activity using both the enzymatic (with the aid of enzymes superoxide dismutase and glutathione peroxidase) and non-enzymatic activity (with ABTS, DPPH and FRAP assay). The antioxidant activity *S. spatulifolius* was performed by Wei et al. [129]. The antioxidant activity of *S. spatulifolius* against acute lung injury in mice induced through lipopolysaccharide was analysed and they found that ethanol extract of the leaf is able to accelerate superoxide dismutase activity in the mice. The analysis revealed the natural free radical scavenging activity and lung protection activity of the plant.

### Anticancer activity

Cancer has become a major threat to the health of human beings. Scientific explorations are going on to discover anti-cancer drugs and medicine combinations to cure cancer [180]. The plant-based natural drugs have also received huge recognition in this exploration and more than one thousand species reported significant anticancer activities. From 1955 onwards, National Cancer Institute (NCI), USA supported the clinical screening of natural compounds for checking anti-cancer properties. Based on that, 114,000 plant-derived compounds were reported and twenty of them got approval for marketing [181]. Chemical compounds such as isoflavones, flavones, flavonoids, coumarins, anthocyanins, catechins, lignans and iso catechinsimpart free radical scavenging activity to plants that is important to tackle cancer [182]. Tetradecanoic acid (myristic acid), 9,12-octadecadienoic acid (Z, Z)- (linoleic acid), 9-octadecenoic acid (Z) (oleic acid), phytol, squalene and acetate are the main compounds from *S. androgynus* which mainly shows cancer-preventive properties [136]. Studies conducted by Rahmat et al. [22] revealed that ethanolic shoot extract of *S. androgynus* shows the inhibiting effects on the proliferation of the cancer cell lines of the breast. The bio-fabricated zinc oxide- *S. androgynus* nanoparticles displayed cytotoxic activity on MDAMB468 human triple-negative breast cancer (TNBC) and NIH3T3 mouse fibroblast cells. Apoptosis of TNBC cells was also observed which indicates the possibility of incorporation of modern technologies to the traditional plants [183]. Therefore it is clear that extension of research on *Sauropus* plants can contribute to cost-effective discovery of anticancer drugs using sustainable approaches.

### Antidiabetic activity

In developing countries, herbal medicines have a key role to play in the dealing with the disease diabetes mellitus [184]. Indian people use *S. androgynus* plant leaves as antidiabetic and for improving vision [6]. The leaves of *S. androgynus* plants have the greater capability to decrease the blood glucose levels of humans which suggest their antidiabetic activity [102]. Indian people use this plant as a leafy vegetable [52]. The scientific evidence of hypoglycemic activity of *S. androgynus* leaves was provided by Suparmi et al. [25]. The streptozotocin-induced diabetes mellitus in male Wistar rats was treated with chlorophyll extracted from *S.androgynus* and it showed a significant hypoglycemic effect. Investigations by Kumar and George [185] also revealed the anti-diabetic activity of *S. androgynus* leaves in alloxan-induced diabetic mice. They found that 250 mg/kg and 500 mg/kg dosage of methanol extract of the leaves leads to the reduction of blood fasting sugar, lipid peroxidation rate and increased antioxidative enzymes such as superoxide dismutase. Since the potential of *S. androgynous* plants is evidenced from the pharmacological studies, they can be used as a natural remedy against this serious metabolic disorder, especially in a scenario, where the number of diabetes patients is predicted to be 439 million adults by 2030.

### Antimicrobial activity

Plants are rich sources of phytochemicals that can be structurally processed and optimised into drugs [186]. Medicinal plants rich in phytochemicals such as alkaloids, terpenes, polyphenols and glycosides show antimicrobial activities [186]. Ethanolic and methanolic extracts of the *S. androgynus* plant show predominant antibacterial activity against *Staphylococcus aureus, Proteus vulgaris* and *Bacillus cereus* [187]. According to Paul and Antos [188], the ethanolic leaf extract of this plant shows potential antibacterial properties against *S. aureus* and *Klebsiella pneumonia*. Methanol leaf extract of katuk shows a higher inhibitory effect against gram-positive bacteria, which is followed by ethanol and aqueous extract [49]. Leaf extract of the plant shows antibacterial activity against *Staphylococcus aureus*, *Bacillus subtilis*, *Escherichia coli* and *Pseudomonas aeruginosa* [189]. The infection of *E. coli* at the time of pregnancy causes hormonal imbalance, hepatic and renal necrosis. The pharmacological study by Christina et al. [190] proved that the combination of *S. androgynus* and *Elephantopus scaber* extract is capable of protecting pregnant mice from *E. coli* induced renal and hepatic necrosis. They have also revealed 75:25 proportion of the two plant extracts are capable of restoring progesterone to normal after the bacteria infection. Husna et al. [191] performed antimicrobial activity of various concentrations (20–100%) of *S. androgynus* against *E. coli* and recorded significant activity. Besides humans, there are several issues faced by the other organisms also. *Vibrio* bacteria is the pathogen that causes vibriosis disease which is creating problems for the shrimp farmers that affect the survival of the shrimp larva. Nurfadillah et al. [43] showed that the 1200 ppm dosage of ethanoic extract of *S. androgynus* treatment generated the highest survival rate (93%) of shrimp larvae infected with vibrio.

There is increasing evidence in favour of the evolution of antimicrobial resistance (AMR) bacterial strains and it is a major challenge at present. Excessive use of antibiotics is one of the reasons behind the widespread issue of AMR. Considering the evolution of new AMR strains, there is growing demand for naturally available sources of compounds that can target AMR bacteria. Interestingly, the extract *S. androgynus* shows antibacterial activity against antibiotic-resistant bacteria such as *Staphylococcus aureus* AMR strain. Rahayu et al. [192] demonstrated the antibacterial activity of the 60% ethanol extract of *S. androgynus* against methicillin-resistant, ampicillin-resistant *Staphylococcus aureus* strain. Bioactive components such as phenolics and flavonoids cause ultrastructural changes in the bacterial membrane which result in their growth reduction. The wounds induced by diabetes are susceptible to *S. aureus* infection, but this organism is resistant to antibiotic methicillin too. Prakoso et al. [193] studied the wound healing capacity of *S. androgynus* alcohol extract in mice with streptozotocin-induced diabetes wounds. The wound was infected with methicillin-resistant *S. aureus* and they recorded a reduction in the infection rate with the extract. The extract was capable of increasing collagen deposition, the tensile strength of skin, skin thickness, vascular endothelial growth factor and decreasing expression of cyclooxygenase-2 expression and C-reactive protein in the mice. If the foods we eat contain natural compounds against the AMR bacteria, it could possibly slow down the issue of AMR.

### Anti-inflammatory

Medicinal plants displayed curation potential against health conditions such as inflammation [194]. Inflammation is the human body’s normal response against pathogen attacks. During inflammation, accumulation of leukocytes occurs and termination of the response is triggered by pro-inflammatory signalling pathways. Failure of this termination mechanism can result in disease or chronic inflammation [195]. The modern drugs used for the treatment of inflammation pose a high risk of cardiovascular diseases and other side effects. The phytochemicals from plants are reported to modulate the pro-inflammatory signal transduction and thereby act as an effective anti-inflammatory agent without side effects [196]. Recently, Kim et al. [197] proved the curating efficiency of *S. brevipes* ethanol extract in gastritis. The oral administration of *S. brevipes* extract (200 mg/kg) reduced the inflammatory lesions in lipopolysaccharide-induced gastritis mice. Zhen et al. [198] proved the anti-inflammatory activity of another species viz. *S. rostratus* which is commonly found in China. Very little information is available on the anti-inflammatory activity of *S. androgynus* and further research should be conducted to prove and validate its anti-inflammatory activity [195].

### Antiobesity activity

The excess accumulation of fat in the body can result in an unhealthy metabolic condition called obesity which further leads to the development of cardiovascular disorders, hypertension, stroke, diabetes mellitus, gallbladder diseases, cancer, and non-alcoholic fatty liver [199]. Therefore prevention of obesity, maintenance of stable and proper body mass index can help in preventing obesity associated diseases [200]. The latest treatment for obesity includes a change in lifestyle with a healthy diet and pharmacological treatments [199]. It was proved that compounds from medicinal plants can be used for inducing weight loss and preventing obesity [23]. *S. androgynus* is commonly used as a slimming agent especially in Taiwan and Malaysia. Its leaf extract is used in order to reduce body weight. *S. androgynous* salads, fries and beverages are taken by many people in the form of antiobesity extracts [55]. The chemical compound that is responsible for the anti-obesity properties in *Sauropus* plants is 3-O-β-D-glucosyl-(1-6)-β-D-glucosyl-kaempferol (GGK) [164]. *S. androgynus* leaf extract in combination with *Zingiber ottensii* rhizome showed a significant rate of adipose tissue protection [201]. Warditiani et al. [202] reported the antidyslipidemic activity (a condition to avoid the occurrence of one or more unhealthy lipids and lipoproteins in the blood). This study found that the saponin extract of *S. androgynus* is capable of reducing the concentrations of triglyceride, total cholesterol, high-density lipoprotein and low-density lipoprotein effectively in the fat-rich diet fed Wistar rats. Therefore it is understood that the inclusion of *Sauropus* leaves in the diet can help to fight against obesity and improve the health of the consumers.

### Lactation inducing activity

Active compounds present in plants have a major role in the postnatal recovery of mothers since the plants have beneficial effects on mother and baby. Study by Ewueke and Chukwu proved that some plants increase lactation in the mother [203]. *S. androgynus* is very popular and it has been extensively used by people in Malaysia especially women for increasing breast milk production [6]. The combination of *Plectranthus amboinicus* and *S. androgynus* can also be used to increase breast milk production [97]. Leaf extracts of this plant aid in lactation [122]. The scientific evidence of lactation-inducing properties of *S. androgynus* is proved not only in humans but also in cattle. Noach et al. [204] proved that cattle feed supplemented with *S. androgynus* leaf powder increases milk production as well as body weight in pregnant cattle and calf respectively. Harjanti et al. [205] observed the positive effect of *S. androgynus*, *Curcuma xanthorrhiza*, and *Alpinia galanga* (70%, 25% and 5% respectively) on milk production and milk quality in mastitis diseased cattle. Their study helped to reveal the therapeutic potential of *S. androgynus* against mastitis. Djati et al. [206] reported prolactin and erythrocyte inducing properties of *S. androgynus* leaves in pregnant mice induced with typhoid using *Salmonella typhi*. These studies prove the galactagogue property of *S. androgynus*.

### Antifungal activity

As discussed, medicinal plants are potential sources of antimicrobial agents [207]. Several medicinal plants are used to treat a variety of fungal infections [208]. *S. androgynus* is traditionally used by Mekong Delta and Central highlands villagers of Vietnam against fungal infection [104]. Its phytochemicals such as flavonoids, tannins, carotenoids and anthocyanins are responsible for its antifungal properties [209]. Neophytadiene found in *S. androgynus* also shows antifungal properties [135].

### Anti-alopecia

Alopecia leads to loss of hair [210]. *S. androgynus* plants can be used for the treatment of alopecia. Topical application of *S. androgynus* mixed with milk induces hair growth in bald people of Kampung Mak Kemas community, Malaysia [211]. Mustarichie et al. [167] demonstrated that 10–25% of ethanol extract-water fraction of *S. androgynus* is capable of inducing hair growth in male rabbits. Anti-alopecia property of *S. androgynus* was performed by Praceka et al. [212] and identified eleven compounds in their study namely, 6-piperidin-1- Ylpyrimidine-2,4-diamine 3-oxide minoxidil, 5-alpha dihydrotestosterone, finasteride, 1,14-tetradecanediol, octadec-1-ene, 1-hexadecene, decanoic acid, phytol, azulene, pyrene and (4s,4’s)-4,4’-(propane-1,3- diyl)bis(3-amino-4-ethyl 1h-pyrazole-5(4h)-one) through computation based molecular modelling.

### Anti-anaemia

Anaemia is a major iron deficiency disorder that particularly affects women and children [213]. Consumption of iron and vitamin rich food can improve anaemia to a certain extent. The use of iron supplement tablets may result in health conditions such as neurogenic disorders, hemochromatosis, and even cancer [214]. Therefore utilisation of natural resources may be attempted to overcome anaemia. Exposure to air pollution can cause reduction in red blood cells and haemoglobin. Siswanto [215] proved that the ethanolic extract of *S. androgynus* is effective against anaemia and increase in RBC and haemoglobin was observed in the mice exposed to motorcycle smoke when treated with ethanolic extract (200 mg/kg weight). Indrayani et al. [39] found that combined extract of *S. androgynous* and *M. oleiefera* raises the haemoglobin and ferritin in rats. Suparmi et al. [25] also obtained similar results with chlorophyll extract of *S. androgynus.* This study observed an increase in *haemoglobin* and ferritin quantity in sodium nitrate induced anaemic mice.

### Antimalarial activity

Malaria is a major disease that affects tropical countries, especially developing countries. Antimalarial activity of *S. androgynus* was explored by Mahardiani et al. [216] and reported significant cytotoxicity against *Plasmodium falciparum* in the Huh7 liver cell lines. Zou et al. [44] revealed the antimalarial activity of another species viz. *S. spatulifolius* (90% of methanol extract) against the pathogen *Plasmodium falciparum*.

### Antitussive effect

Antitussive medicines have wide popularity all over the world due to the prevalence of cough [26]. Natural bioactive compounds contribute significantly to the relief of cough and cold [217]. Several studies have explored the antitussive activity of herbal medicines [26, 218–220]. *S. spatulifolius* reported remarkable antitussive activity against ammonia-liquor induced cough in mice with 75% ethanol and ethyl acetate extract. The study was focused on the effect of the extracts on ATP-sensitive potassium channels and opioid receptors and it showed positive activity [24].

### Analgesic activity

The actual or potential tissue damage can result in pain [221]. The most popular narcotic drugs used for relief from pain are associated with several side effects [222]. So, there exists a demand to develop analgesic drugs from traditional medicinal and other phytochemical resources [221]. The analgesic activity of the *S. rostratus* was analysed with acetic acid-induced pain and hot plate induced heat simulation on mice. It was found that the aqueous extract of the plant has the capacity to reduce pain twists in mice [198]. Selvi et al. [47] also reported the analgesic activity of *S. androgynus* leaf extract in the rats where a hot plate test was applied to induce pain and dese dependent inhibition of hot plate reaction was observed.

### Antiulcer activity

The anti-ulcer characteristics of *S. androgynus* was revealed by Roosdiana et al. [45]. The ethanolic extract of *S. androgynus* was used for the treatment of peptic ulcers in rats in an aspirin-induced model. It was found that 48.6 mg/200 g BW was the most efficient dose in the rats and it induced the tissue repair protein tumour necrosis factor-alpha (TNF-α) at the time of inflammation (Table 6).

**Table 6:** Pharmacological potential of *S. androgynus*

Activity	Part used; Extract; Application; Animal/cell lines	Animal model/In vitro study (Dose); Reference control; Activity (Reference)	Toxicity	References
Antibacterial activity	Leaf; ethanol extract; i.p. administration; vannamei shrimp larvae (0.01 g)	*Vibrio* sp.; 600, 800, 1000, 1200 ppm of *Sauropus* leaf extract; w/o reference	NA	[43]
	Leaf; ethanol and aqueous; *Klebsiella pneumoniae* and *Staphylococcus aureus*	Disc diffusion method; 0.04 mg/ml; gentamicin	NA	[188]
	Leaf; aqueous, ethanol and methanol; *Bacillus subtilis*, *B. cereus*, *S. aureus*, *E. coli*, *K. pneumoniae*, *S. typhimurium*	Disc diffusion method; Streptomycin	NA	[49]
	Stem; methanol; *Escherichia coli, Klebsiella sp., Edwardsiella tarda, Flavobacterium sp., Aeromonas hydrophila, Salmonella sp., Pseudomonas aeruginosa, Vibrio alginolyticus, V. cholerae and V. parahaemolyticus*	Broth microdilution method; 1 mg/ml	NA	[129]
	Callus and leaf; methanol; *E. coli, B. subtilis, S. aureus, S. typhi*	Disc diffusion method	NA	[223]
Antioxidant activity	Leaf; ethanol; intraperitoneal injection (i.p.); male Wistar rats (seven weeks old)	Dissolved in 0.5 to 1.0 mg Coenzyme Q10 (CoQ10) /kg/day; CoQ10 supplement	Safe up to 1.0 mg CoQ 10/kg/day	[42]
	Chlorophyll extract in methanol:acetone; oral; Wistar albino rats (150–200 g)	NaNO_2_ induced oxidative stress; 0.016 mg/ml and 0.008 mg/ml; Cu-chlorophyllin (0.008 mg/ml)	NA	[224]
Galactagogue activity	Leaf; aqueous; oral administration; Wistar rats	Lactating rats; 26.25, 52.5 and 105 mg/kg/day; domperidone (2.7 mg/kg/day); distilled water	Safe up to 105 mg/kg/day	[40]
	Leaf; ethanol; oral administration; Etawah goat (38.19 ± 2.4 kg)	Concentrate containing katuk leaf flour and bio complex Zn flour; w/o reference	NA	[204]
Digestibility	Leaf; oral administration; Friesian Holstein cattle (300–450 kg)	155 g katuk leaf and 15% of Gamal leaves; w/o reference	NA	[41]
Hepatic necrosis	Leaf; ethanol; i.p. administration; BALB/c mice (20–25 g)	*E. coli*- infected pregnant mice model; 37.5, 75, 50, 112.5, 150 mg/kg SA extract with *E. scaber* extract; w/o reference	Safe up to the mentioned concentration	[190]
Iron deficiency anaemia	Leaf; ethanol; oral administration; female Wistar strain rats (190–220 g)	Iron deficient diet; 300 mg/day; w/o reference	Safe up to 300 mg/g	[39]
	Leaf methanol; acetone extract; oral; Female mice, Balb-c	Sodium nitrile induced anaemia; Cu-chlorophyllin (13.29, 11.83, 14.54, 13.99 g/dl)	NA	[215]
	Leaf; methanol: acetone; IDA female rats (150–200 g)	Iron deficient diet for 14 days; 0.016 mg/mL; commercial chlorophyll (0.016 mg/mL)	Safe up to 0.016 mg/ml	[25]
Teratogenic effects	Leaf; methanol; i.p. administration; Zebrafish	10, 100, 1000 mg/mL leaf extract and 1% dimethyl sulfoxide; w/o reference	Safe up to 1000 mg/mL	[225]
	Leaf powder; oral; Hainan in rats	Pregnant mice; 0, 250, 500, 1000 mg/kg	NA	[226]
Antidiabetic activity	Leaf; ethanol; i.p. administration; Wistar albino rats; (180–220 g)	Alloxan induced diabetes; 200 and 400 mg/kg; glibenclamide (10 mg/kg)	Safe up to 400 mg/kg	[52]
	Leaf; acetone, methanol (7:3); oral; Wistar rats (150–200 g)	Streptomycin induced diabetes; 0.016 mg/mL chlorophyll extract; glibenclamide (0,09 mg/200)	0.016 mg/mL concentration of chlorophyll extract was low toxic to the internal organs of rats	[25]
	Leaf; methanol; oral; mice	Alloxan induced diabetes; 250 mg/ kg and 500 mg/kg; glibenclamide (2.5 mg/kg)	Non-toxic up to 500 mg/kg	[185]
Anticancer activity	Leaf; ethanol; breast cancer cell line breast cancer (MDA-MB-231 and MCF-7)	3-(4,5-dimethylthiazol-2-yl)-2,5-diphenyltetrazolium bromide (MTT) assay; 5, 10, 20, 40, 60, 80 and 100 µg/ml	Safe up to 100 µg/ml	[22]
Antiinflammatory activity	Leaf; ethanol; ip; albino mice (20–25 g)	Carrageenan induced rat paw edema; 100, 200 and 400 mg/kg BW; Phenylbutazone (100 mg/kg bw)	Nontoxic up to 400 mg/kg	[47]
	Leaf (*S. brevipes*); ethanol; oral; mice	Lipopolysaccharide induced peritonitis; 200 mg/kg; Ranitidine (40 mg/kg)	Nontoxic up to 200 mg/kg	[197]
	Leaves; aqueous protein extract; erythrocytes	Hypotonic solution-induced hemolysis; 10, 25, 50, 100 μg/mL; acetylsalcylic acid (100 μg)	NA	[60]
	Leaf; ethanol; transdermal; male Wistar rat	Carrageenan induced; 400 mg / kg BW	NA	[227]
Anti-obesity activity	Leaf; ethanol; oral; white male rat (200–300 g)	High fat diet; 50, 75, 100 mg/kg bw; Orlistat and metformin	Nontoxic up to 100 mg/kg	[201]
Anti-alopecia activity	Leaf; ethanol; topical; male rabbit (1.5–2 kg)	Hair length measurement; Minoxidil (2%)	NA	[167]

## Toxicity of *Sauropus androgynus*

Several studies showed that regular consumption of *S. androgynus* leads to obstructive ventilatory impairment especially in patients with respiratory symptoms [6, 228]. Overconsumption of papaverine is known to cause bronchiolitis obliterans [6]. Since *S. androgynus* contains papaverine, further studies were conducted to investigate its role in bronchiolitis obliterans disease [20]. In Taiwan and Japan, young and middle-aged women who consumed *S. androgynus* extracts for weight control, later developed bronchiolitis obliterans [229, 230]. Similar findings were also obtained by Ger et al. [231] suggesting potential side effects of *S. androgynus*. Yu et al. [98] observed the cytotoxic effect of *S. androgynus* in mouse embryonic fibroblast NIH3T3 cells*.* The ethyl acetate (EtOAc) extract of the plants recorded the highest inhibition of cell growth and the results reveal apoptosis and necrosis effects of the *S. androgynus* extract [98]. Papaverine at a dose of 200 mg/day is used as antispasmodic, but if it is taken in larger amounts can result in constipation, drowsiness and increased reflex excitability [232]. Hsiue et al. [233] found that people in Taiwan who consumed *S. androgynus* in higher amounts developed minor to severe obstructive ventilatory problems within 6–7 months and it was very common in people who ingested more than 3600 g. Ou et al. [234] reported that there is no long-term effect of the *S. androgynus* on lung diseases. Chronic ingestion of katuk extract more than 131 g/day is associated with insomnia, weight loss, breathing difficulty, poor appetite, palpitation, cough, skin rashes and dizziness [235]. Omar et al. [225] observed the teratogenic activity of *S. androgynus* (10–1000 µg/mL) in the zebrafish. Therefore it is very important to consider the toxicological properties of the plant while preferring for consumption and disease treatments.

## Conclusions

*Sauropus androgynus* is an important green leafy vegetable with high nutritional and therapeutic potential. This comprehensive review discusses the multiple ethnic dishes obtained from it and its use in ethnomedicinal preparations. Extensive review of literature shows that it is a multipurpose plant with uses in various ethnic food and ethnomedicinal preparations. It has a very high regional importance among the people in South, East and Southeast Asia. Documentation of regionally available ethnic food systems and investigation of their dietary contributions is crucial in the present context considering the recurrent lockdowns and disruptions of the supply chains in long distance food transportation. The presence of various phytochemicals such as phenols, terpenoids, tannins, steroids, alkaloids, fatty acids, flavonoids, and volatile oils makes it a very important medicinal and edible herb. These compounds might be responsible for its bioactivities as revealed by pharmacological studies. It has been pharmacologically demonstrated that *S. androgynus* have significant antioxidant, anti-cancerous, antidiabetic, antimicrobial, and anti-inflammatory, anti obesity, and lactation inducing, antifungal, anti-alopecia, anti-anaemia, antitussive, analgesic and antiulcer activities. But further extensive in vivo and clinical studies are required to validate its medicinal activities. Several toxicological effects such as bronchiolitis obliterans, insomnia, weight loss, dizziness and teratogenic activity of *S. androgynus* provide an idea about its potential side effects*.* The future studies should be focused on the investigation of more bioactive compounds and their potential roles in treating diseases. Isolation of some of the pure compounds and developing drugs from them is also an important area of research. Scientific research on its cultivation, development of varieties that are nutritionally superior should also be attempted. The recent research shows the importance of the regionally relevant local traditional/ethnic food systems. Therefore, this comprehensive review on the ethnic foods that are prepared from *S. androgynus* is very timely and more such studies should be attempted to document the plant based ethnic food systems from various parts of the world. The contributions of the regional ethnic food systems to the food security and food system resilience in times of situations such as pandemics should be empirically demonstrated to devise future strategies aimed at strengthening the local food systems.

## Data Availability

All material/data used are available in the manuscript.

## Abbreviations

BO: Bronchiolitis obliterans
DPPH: 2,2-Diphenyl-1-picryl-hydrazyl-hydrate
EtOAc: Ethyl acetate
FRAP: Ferric reducing antioxidant power assay
FTIR: Fourier Transform Infrared spectroscopy
GC–MS: Gas Chromatography–Mass Spectroscopy
GGK: 3-O-β-D-glucosyl-(1-6)-β-D-glucosyl-kaempferol
RBC: Red blood cells
SA: *Sauropus androgynus*
TNBC: Triple negative breast cancer
